# Pharmacological activation of AMPK and glucose uptake in cultured human skeletal muscle cells from patients with ME/CFS

**DOI:** 10.1042/BSR20180242

**Published:** 2018-05-08

**Authors:** Audrey E. Brown, Beth Dibnah, Emily Fisher, Julia L. Newton, Mark Walker

**Affiliations:** 1 Institute of Cellular Medicine, Newcastle University, Newcastle upon Tyne, U.K.; 2 Clinical Academic Office, Newcastle upon Tyne Hospitals NHS Foundation Trust, Newcastle upon Tyne, U.K.

**Keywords:** AMPK, glucose uptake, muscle contraction

## Abstract

Skeletal muscle fatigue and post-exertional malaise are key symptoms of myalgic encephalomyelitis (ME)/chronic fatigue syndrome (ME/CFS). We have previously shown that AMP-activated protein kinase (AMPK) activation and glucose uptake are impaired in primary human skeletal muscle cell cultures derived from patients with ME/CFS in response to electrical pulse stimulation (EPS), a method which induces contraction of muscle cells *in vitro*. The aim of the present study was to assess if AMPK could be activated pharmacologically in ME/CFS. Primary skeletal muscle cell cultures from patients with ME/CFS and healthy controls were treated with either metformin or compound 991. AMPK activation was assessed by Western blot and glucose uptake measured. Both metformin and 991 treatment significantly increased AMPK activation and glucose uptake in muscle cell cultures from both controls and ME/CFS. Cellular ATP content was unaffected by treatment although ATP content was significantly decreased in ME/CFS compared with controls. Pharmacological activation of AMPK can improve glucose uptake in muscle cell cultures from patients with ME/CFS. This suggests that the failure of EPS to activate AMPK in these muscle cultures is due to a defect proximal to AMPK. Further work is required to delineate the defect and determine whether pharmacological activation of AMPK improves muscle function in patients with ME/CFS.

Chronic fatigue syndrome (CFS), also known as myalgic encephalomyelitis (ME), is a long-term illness affecting approximately 250000 people in the U.K., and has a wide range of symptoms. The principal symptom is unexplained persistent fatigue, lasting for at least 6 months, which cannot be alleviated by rest [1]. This is often accompanied by cognitive deficits, muscle weakness and sleep disturbances. Post-exertional malaise, where symptoms are amplified or prolonged after minor exertion, is another key symptom of ME/CFS [2].

AMP-activated protein kinase (AMPK) is a key mediator of the skeletal muscle response to exercise [3]. In response to an energy deficit, such as during muscle contraction, AMPK is activated resulting in the switching off of ATP-consuming processes, and the switching on of ATP-generating processes. In skeletal muscle, this includes an increase in the uptake of glucose into the muscle cell [4]. We have previously shown that AMPK activation is impaired in primary human skeletal muscle cell cultures derived from patients with ME/CFS in response to electrical pulse stimulation (EPS), a method which induces contraction of muscle cells *in vitro* [5]. This abnormality in AMPK activation also resulted in a failure to increase glucose uptake into the cell in response to EPS.

AMPK is a heterotrimeric complex of α, β and γ subunits. Multiple isoforms exist for each subunit and some isoforms may be expressed in a cell-type or tissue-specific manner [6,7]. AMPK can be activated allosterically, particularly by AMP or via activation of upstream kinases including liver kinase B1 (LKB1) and the Ca^2+^/calmodulin-dependent kinase kinase (CaMKK) [8]. AMPK can also be regulated by pharmacological agents including metformin, which activates AMPK indirectly by inhibiting ATP synthesis. A number of small molecule activators have been developed which bind directly to AMPK, resulting in allosteric activation of AMPK [9].

The aim of the present study was to examine if AMPK can be modified by pharmacological treatment in primary human skeletal muscle cell cultures derived from patients with ME/CFS. We used both an indirect activator of AMPK (metformin) and a direct activator (Compound 991) to assess AMPK activation, glucose uptake and ATP content of muscle cells.

## Materials and methods

### Study subjects

Muscle biopsies were obtained from 10 patients diagnosed with CFS and 7 healthy control subjects. Groups were matched for age and comprised males and females. All subjects were recruited via the Newcastle NHS CFS Clinical Service at the Newcastle Hospitals NHS Foundation Trust. All subjects fulfilled the Fukuda criteria [1] and provided written informed consents. None had evidence of neurological deficit based on clinical assessment. The study was approved by the Newcastle and North Tyneside Joint Ethics Committee.

### General chemicals and reagents

Cell culture medium was obtained from Scientific Laboratory Supplies (U.K.). FBS and trypsin-EDTA were obtained from Life Technologies (Paisley, U.K.). Chick embryo extract was purchased from Sera Labs International (Sussex, U.K.). p-AMPK^Thr172^ (40H9) and total AMPKα (F6) antibodies were obtained from New England Biolabs (Herts, U.K.) while β-actin (clone AC-15) was purchased from Sigma. Monoclonal mouse antihuman desmin (D33) antibody was obtained from DAKO. Vector VIP HRP-substrate kit was obtained from Vector Laboratories. 2-Deoxy-D-[2,6-^3^H]glucose was purchased from Hartmann Analytic (Germany). Compound 991 was donated by AstraZeneca. Metformin was obtained from Sigma.

### Cell culture

Muscle biopsies were obtained from the vastus lateralis and muscle precursor cells isolated as described in detail previously [5,10]. Myoblasts were cultured in Ham’s F10 medium supplemented with 20% (v/v) FBS and 2% (v/v) chick embryo extract. At confluence, differentiation was induced by changing the media to minimal essential media supplemented with 2% (v/v) FBS. All experiments were performed on day 7 differentiated myotubes, passage 7.

### Western blotting

Cells were lysed in extraction buffer (100 mM Tris/HCl, pH 7.4, 100 mM KCl, 1 mM EDTA, 25 mM KF, 1 mM benzamidine, 0.5 mM Na_3_VO_4_, 0.1% (v/v) Triton X-100, 1× protease inhibitor cocktail (Pierce) before sonicating for 10 s). Protein concentrations were determined spectrophotometrically at 595 nm by a Coomassie binding method (Pierce). Ten microgram samples were prepared in Laemmli sample buffer (0.125 M Tris/HCl, pH 6.8, 4% (w/v) SDS, 20% (v/v) glycerol, 10% (v/v) 2-mercaptoethanol and 0.004% (w/v) Bromophenol Blue) and boiled for 5 min. After separation on SDS/PAGE (10% gels), proteins were transferred on to PVDF membranes using a mini-Hoeffer wet transfer system. After incubation with the appropriate antibodies, detection took place using ECL. p-AMPK antibody was used at 1:1000 dilution while AMPKα was used at 1:2000 dilution. β-actin was used at 1:10000. Densitometry was performed using a Bio-Rad Molecular Imager GS-800 calibrated densitometer and Quantity One software. Depending upon the exposure time, the AMPKα detected more than one isoform. Under these circumstances, the band corresponding to the p-AMPK band was used to for densitometric analysis. All antibodies were obtained from New England Biolabs.

### ATP assay

Cellular ATP content of cells was assessed by ATP luciferase assay. After the indicated treatment times, medium was removed and cells extracted in 0.1 M NaOH solution. A standard curve was generated using 1 mM ATP (Sigma) diluted in 0.1 M NaOH and 20 µl each standard and sample were added in duplicate to a 96-well white luminescence plate. A 50-µl aliquot of FL-AAM assay mix (Sigma) was added to 10 ml ATP assay buffer (0.1 M Tris, pH 7.75, 10 mM Mg-acetate, 1.8 mM EDTA, 0.6 g/l BSA) and 100 µl this solution was added to each well of the 96-well plate. Luminescence was measured immediately and ATP content determined relative to the ATP standard curve. Protein content of the extracted samples was measured spectrophotometrically at 595 nm by a Coomassie binding method (Pierce).

### Glucose uptake

Glucose uptake was measured by incubating cells in tritiated 2-deoxyglucose after treatment with either metformin or compound 991. After treatment, cells were incubated in Krebs’ buffer (136 mM NaCl, 4.7 mM KCl, 1.25 mM MgSO_4_, 1.2 mM CaCl_2_, 20 mM HEPES, pH 7.4) with or without 100 nM insulin or cytochalasin B (10 μM) for 20 min; 0.1 mM 2-deoxyglucose and 0.5 μCi (2,6-^3^H) 2-deoxyglucose were added to each well and incubated for a further 10 min. The reaction was stopped by washing the plate rapidly in ice-cold PBS. Cells were lysed in 0.05% SDS before scintillation counting and protein determination. The specific activity of the tritiated glucose was calculated before it was added to the cells and this was used to calculate the nmol glucose incorporated into the cells over 1 min. Data were normalized to total protein content and presented as pmol/min/mg.

### Statistical analysis

All results are expressed as mean ± S.E.M. unless where stated. Data were analysed using one-way or two-way ANOVA where appropriate and, where significant, followed up by *t*test between groups. Statistical analyses were performed using GraphPad Prism (California) software.

## Results

### Effect of metformin and 991 on AMPK activation

**Figure 1:**
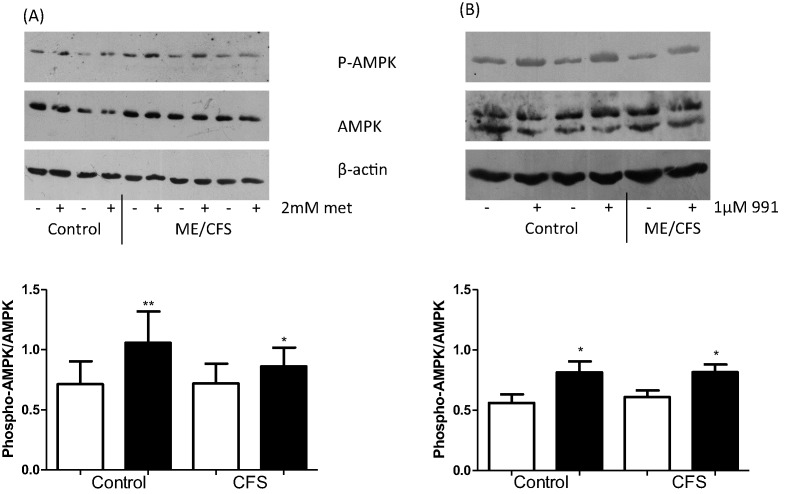
Phosphorylation of AMPK^Thr172^ was assessed by Western blot of myotubes after treatment (**A**) 2 mM metformin for 16 h (**B**) 1 µM 991 for 2 h. Densitometry is expressed as a ratio of phosphorylated to total protein. Open bar: untreated, closed bar: treated. *P<0.05, **P<0.01. *n*=7 controls and 8 CFS.

Skeletal muscle cells from seven healthy controls and eight CFS subjects were grown to confluence and allowed to differentiate for 7 days. Cells were then treated with either 2 mM metformin for 16 h or 1 µM 991 for 2 h prior to protein extraction and Western blotting. Figure 1A shows that metformin treatment significantly increased AMPK activation, as measured by phosphorylation of AMPK on residue Thr^172^, in both control (*P*<0.01) and CFS (*P*<0.05) myotubes, compared with untreated. Compound 991 treatment had a similar effect, significantly increasing AMPK activation over untreated myotubes in both control and CFS (*P*<0.05, both) (Figure 1B). ACC phosphorylation increased in a dose-dependent manner in response to compound 991 (see Supplementary Figure S1).

### Effect of metformin and 991 on glucose uptake

**Figure 2:**
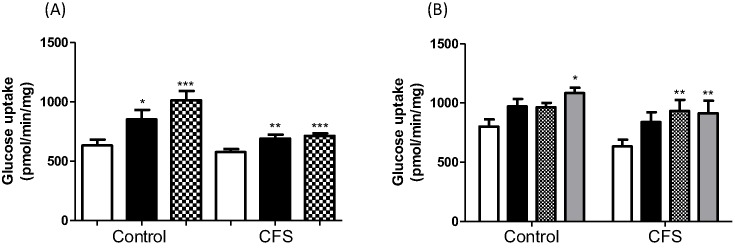
Glucose uptake was measured in control and CFS myotubes after treatment (**A**) 2 mM metformin for 16 h. Open bar: untreated, closed bar: insulin treatment, hatched bar: metformin treatment. (**B**) 0.1 and 1 µM 991 for 2 h. Open bar: untreated, closed bar: insulin treatment, hatched bar: 0.1 µM 991, grey bar: 1 µM 991. *P<0.05, **P<0.01, ***P<0.0005. *n*=7 controls and 8 CFS assayed at least in duplicate.

Activation of AMPK would be expected to lead to an increase in glucose uptake. Figure 2A shows that metformin treatment significantly increased glucose uptake in both control and CFS cells, and the effect was comparable with that of insulin. In controls, metformin increased glucose uptake from 632.8 ± 50.4 to 1014 ± 79.2 pmol/mg/min (*P*<0.0005), while in CFS, glucose uptake increased from 576.4 ± 26.5 to 715 ± 21.2 pmol/mg/min (*P*<0.0005). Compound 991 treatment also increased glucose uptake at concentrations of 0.1 and 1 µM in both control and CFS cells (Figure 2B). In controls, glucose uptake increased from 802.2 ± 59.4 to 963.9 ± 37.9 pmol/mg/min and 1084.3 ± 44.9 pmol/mg/min (*P*<0.05) for 0.1 and 1 µM 991, respectively. Compound 991 increased glucose uptake from 633.8 ± 56.8 to 933.7 ± 93.3 pmol/mg/min (*P*<0.01) and 913.9 ± 105.7 pmol/mg/min (*P*<0.01) for 0.1 and 1 µM, respectively. In both control and CFS cells, the effect of 991 treatment was comparable with that of insulin.

### Effect of metformin and 991 on cellular ATP content

**Figure 3:**
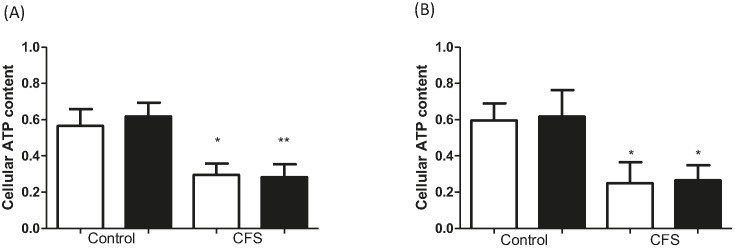
Cellular ATP content measured in control and CFS myotubes after treatment (**A**) 2 mM metformin for 16 h. (**B**) 1 µM 991 for 2 h. Open bar: untreated, closed bar: treated. Data were normalized to untreated. *P<0.05, **P<0.01. *n*=7 control and 8 CFS assayed in duplicate.

Treatment with either metformin or 991 did not reduce cellular ATP content (Figure 3). However, ATP content is significantly decreased in muscle cell cultures from CFS compared with control (*P*<0.05), regardless of treatment.

## Discussion

The present study demonstrates that AMPK and subsequent downstream effects can be activated by both an indirect (metformin) and direct (compound 991) activator of AMPK in skeletal muscle cells from patients with ME/CFS. This is in contrast with our previous study of the same muscle cell cultures which showed that EPS-mediated contraction failed to activate AMPK and glucose uptake in skeletal muscle cells from patients with ME/CFS [5]. Together, these findings point to a signalling defect proximal to AMPK, and further studies are underway to explore the expression and function of the key proximal signalling molecules in the ME/CFS cultures. This is the first report to show that 991 is active in human skeletal muscle cell cultures. Compound 991 has previously been shown to increase AMPK activity and glucose uptake in isolated rat skeletal muscle [11]. These effects are ablated in AMPKα1-/α2 knockout mouse myotubes, suggesting that 991 acts specifically via AMPK. Further evidence for the specificity of 991 for AMPK is indicated by a screen of 991 against a panel of protein kinases in cell-free assays. This showed that 991 enhances AMPK activity while it has no effect on upstream kinases including LKB1 and CaMKK [12].

Current evidence indicates that AMPK activation increases glucose uptake through activation of AS160 and glucose transporter translocation to the cell membrane [13]. As part of our ongoing work, we are exploring the fate of the AMPK-mediated glucose uptake with a particular focus on glucose oxidation and glycolysis in the ME/CFS cultures. The regulation of AMPK is complex. As well as allosteric activation by the binding of AMP, AMPK can be activated by upstream kinases such as LKB1 and CaMKK. Impaired exercise-stimulated glucose uptake in muscle-specific LKB1 knockout mouse models suggests that LKB1 may be the predominant kinase involved in regulation of AMPK by contraction in skeletal muscle [14,15]. The relationship between AMPK and exercise tolerance has also been demonstrated in AMPK-knockout mouse models. A skeletal muscle-specific AMPK knockout model demonstrated a reduced exercise capacity, as assessed by voluntary wheel running and treadmill running to exhaustion. Impaired muscle function was indicated by a reduction in maximal force and fatigue resistance *ex vivo* [16]. It is also clear that muscle function is closely dependent on metabolic function, particularly glucose uptake, as shown in GLUT4 knockout mouse models. These models show that when glucose uptake is impaired, susceptibility to fatigue in response to exercise is enhanced [17,18]. Gorselink et al. [17] also showed that peak power output and contractile performance were reduced in these mouse models. As observed in our ME/CFS skeletal muscle cell cultures, in response to contraction induced by EPS, both AMPK activation and glucose uptake is impaired [5]. We would predict that the lack of effect on glucose uptake by EPS would impair muscle contractile function, leading to exercise intolerance. The current study suggests that this failure to activate AMPK in response to contraction could be bypassed by pharmacological intervention, and contributes to the evidence base for a clinical trial of an AMPK activator in ME/CFS.

Another key finding from the present study is the significant decrease in cellular ATP content in skeletal muscle from ME/CFS compared with healthy controls. This decrease occurred regardless of treatment and was measured independently on two separate occasions. Reduced ATP content has previously been observed post-exercise in ME/CFS patients [19] and more recently, impaired ATP synthesis has been observed *in vivo* in patients [20]. ATP content influences cell survival, with an age-related decrease in ATP content in cultured fibroblasts being linked with an increased susceptibility to cell death by necrosis [21]. However, another study recently reported increased ATP levels in peripheral blood mononuclear cells from patients with ME/CFS [22]. These authors suggested that fatigue may be linked to non-mitochondrial processes which produce ATP, such as glycolysis.

Potential causes of reduction in cellular ATP content include impairment in mitochondrial function, a decrease in mitochondrial membrane potential or increased reactive oxygen/nitrogen species (ROS/RNS) production. Evidence for a decrease in skeletal muscle mitochondrial function in ME/CFS is conflicting. Some studies have found a decreased mitochondrial content, but not function in skeletal muscle biopsies from ME/CFS patients compared with healthy controls [23], while others have identified metabolic abnormalities in ME/CFS consistent with impaired mitochondrial function [24]. ROS may play an important role in the ability of skeletal muscle to adapt to exercise but equally, increased ROS can impair mitochondrial function, reduce muscle contractile force, and contribute to muscle dysfunction [25]. There is some evidence to suggest that oxidative stress pathways are activated *in vivo* in ME/CFS [26,27] but, to our knowledge, this has not been examined *in vitro*. Future work will focus on exploring the mechanisms by which contraction fails to activate AMPK and on mitochondrial function in skeletal muscle.

The important findings from the present study are that, firstly, pharmacological activation of AMPK can improve glucose uptake in skeletal muscle cell cultures from patients with ME/CFS and secondly, cellular ATP content is significantly reduced in ME/CFS muscle cell cultures. The observation that AMPK was activated directly by metformin and 991 but not EPS in the ME/CFS cultures points to a signalling defect proximal to AMPK. Further work is required to delineate the defect and determine whether pharmacological activation of AMPK improves muscle function in patients with ME/CFS.

### Supporting information

**Supplementary Figure:** Control myotubes treated with Compound 991 at 0, 0.001, 0.01,0.1, 1, 10μM. Top blot is Phospho-ACC and bottom blot is β-actin

## Abbreviations

ACC: Acetyl-CoA Carboxylase
AMPK: AMP-activated protein kinase
CaMKK: Ca^2+^/calmodulin-dependent kinase kinase
CFS: chronic fatigue syndrome
EPS: electrical pulse stimulation
GLUT4: Glucose transporter 4
LKB1: liver kinase B1
ME: myalgic encephalomyelitis
