# Supplementary material for: Pharmacological activation of AMPK and glucose uptake in cultured human skeletal muscle cells from patients with ME/CFS
Source: Biosci Rep. 2018 May 8;38(3):BSR20180242. doi: 10.1042/BSR20180242 (PMC5938427; doi:10.1042/BSR20180242)
Supplement: Supplementary file 1 [file bsr20180242_Supp1.pdf]

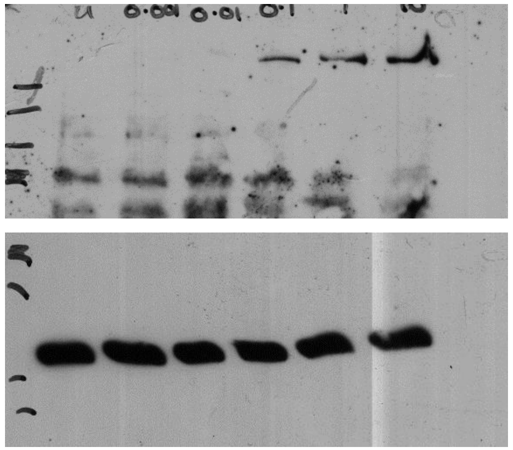

Supplementary Figure. Control myotubes treated with Compound 991 at 0, 0.001, 0.01,0.1, 1, 10μM. Top blot is Phospho-ACC and bottom blot is β-actin
